# A symmetric prior knowledge based deep learning model for intracerebral hemorrhage lesion segmentation

**DOI:** 10.3389/fphys.2022.977427

**Published:** 2022-11-23

**Authors:** Mayidili Nijiati, Abudouresuli Tuersun, Yue Zhang, Qing Yuan, Ping Gong, Abudoukeyoumujiang Abulizi, Awanisa Tuoheti, Adili Abulaiti, Xiaoguang Zou

**Affiliations:** ^1^ Department of Radiology, The First People’s Hospital of Kashi Prefecture, Kashi, China; ^2^ Deepwise AI Lab, Beijing, China; ^3^ Clinical Medical Research Center, The First People’s Hospital of Kashi Prefecture, Kashi, China

**Keywords:** intracerebral hemorrhage, lesion segmentation, deep learning, symmetric knowledge, transformer

## Abstract

**Background:** Accurate localization and classification of intracerebral hemorrhage (ICH) lesions are of great significance for the treatment and prognosis of patients with ICH. The purpose of this study is to develop a symmetric prior knowledge based deep learning model to segment ICH lesions in computed tomography (CT).

**Methods:** A novel symmetric Transformer network (Sym-TransNet) is designed to segment ICH lesions in CT images. A cohort of 1,157 patients diagnosed with ICH is established to train (*n* = 857), validate (*n* = 100), and test (*n* = 200) the Sym-TransNet. A healthy cohort of 200 subjects is added, establishing a test set with balanced positive and negative cases (*n* = 400), to further evaluate the accuracy, sensitivity, and specificity of the diagnosis of ICH. The segmentation results are obtained after data pre-processing and Sym-TransNet. The DICE coefficient is used to evaluate the similarity between the segmentation results and the segmentation gold standard. Furthermore, some recent deep learning methods are reproduced to compare with Sym-TransNet, and statistical analysis is performed to prove the statistical significance of the proposed method. Ablation experiments are conducted to prove that each component in Sym-TransNet could effectively improve the DICE coefficient of ICH lesions.

**Results:** For the segmentation of ICH lesions, the DICE coefficient of Sym-TransNet is 0.716 ± 0.031 in the test set which contains 200 CT images of ICH. The DICE coefficients of five subtypes of ICH, including intraparenchymal hemorrhage (IPH), intraventricular hemorrhage (IVH), extradural hemorrhage (EDH), subdural hemorrhage (SDH), and subarachnoid hemorrhage (SAH), are 0.784 ± 0.039, 0.680 ± 0.049, 0.359 ± 0.186, 0.534 ± 0.455, and 0.337 ± 0.044, respectively. Statistical results show that the proposed Sym-TransNet can significantly improve the DICE coefficient of ICH lesions in most cases. In addition, the accuracy, sensitivity, and specificity of Sym-TransNet in the diagnosis of ICH in 400 CT images are 91.25%, 98.50%, and 84.00%, respectively.

**Conclusion:** Compared with recent mainstream deep learning methods, the proposed Sym-TransNet can segment and identify different types of lesions from CT images of ICH patients more effectively. Moreover, the Sym-TransNet can diagnose ICH more stably and efficiently, which has clinical application prospects.

## Introduction

Intracerebral hemorrhage (ICH) is one of the most devastating subtypes of stroke, accounting for 10%–20% of all stroke cases (Zhao et al., 2020). ICH is commonly caused by trauma, hypertension, and vascular malformation, and more than half of the ICH patients have a long-term disabilities (Rindler et al., 2020). According to symptom onset time, the patients are divided into hyperacute stage (≤6 h), acute stage (7–72 h), subacute stage (3 days–2 weeks), and chronic stage (after 2 weeks) (Vijayan and Reddy, 2016). Depending on the hemorrhage site, ICH can be divided into five types, which include intraparenchymal hemorrhage (IPH), intraventricular hemorrhage (IVH), extradural hemorrhage (EDH), subdural hemorrhage (SDH), and subarachnoid hemorrhage (SAH) (Chilamkurthy et al., 2018). Different bleeding types determine the treatment plan and prognosis of patients. Therefore, accurate detection and classification of ICH are of great significance for saving the life and neurological function of patients (Li et al., 2021).

Neuroimaging is an important tool for the detection, characterization, and prediction of acute stroke, including ischemic and hemorrhagic subtypes (Lee et al., 2019). Computed tomography (CT) is the first choice for emergency diagnosis of ICH due to its high imaging speed (Chan, 2007). However, reading and analyzing a large amount of CT images is time-consuming and tricky work for clinic doctors, which increases the possibility of missed diagnosis and misdiagnosis (Cho et al., 2019). At present, emergency craniocerebral CT diagnosis, especially on the night shift, is mostly provided by the junior radiologist, and then reviewed by the senior radiologist (Lal et al., 2000; Erly et al., 2002). Several studies have shown that initial diagnosis provided by junior radiologists has different degrees of missed diagnosis and misdiagnosis (Erly et al., 2002). However, due to the high variability of the location, contrast, and shape of bleeds, accurate localization of them can be challenging and time-consuming even for experienced radiologists. In addition, due to limited medical conditions and resources in some underdeveloped areas, patients with ICH cannot receive an accurate diagnosis and timely treatment the first time, resulting in a threat to patients life. Therefore, it is very important to diagnose and classify ICH timely and accurate (Li et al., 2021).

Artificial intelligence (AI) technology is a rapidly developing field, which is regarded as a promising approach for fast and efficient image analysis (He et al., 2016; Li Y. et al., 2022). In recent years, AI has been applied in the medical imaging field of acute cerebrovascular diseases, including as a tool for classification, quantification, monitoring, and prediction (Ironside et al., 2019; Sun et al., 2020; Zhu et al., 2020). The convolutional neural network (CNN) is one of the representative deep learning algorithms that utilize image high-dimensional digital information by extracting image features (Badrinarayanan et al., 2017; Roy et al., 2019). In the field of medical image segmentation, the U-shaped network (U-Net) is one of the most representative convolutional neural networks (Ronneberger et al., 2015). In several years, many deep learning methods based on convolutional neural networks have been successfully applied to ICH lesion segmentation and achieved relatively ideal results. Inkeaw et al. proposed a 3D convolutional neural network, which processes CT images with different resolutions through four parallel paths, and segments different types of bleeding lesions through the region-growing method. The median DICE coefficient of segmentation for each bleeding subtype was higher than 0.37 (Inkeaw et al., 2022). Xu et al. (2021) adopted the densely connected U-Net architecture to test on nearly 300 ICH images and achieved a DICE coefficient of 0.89. Nevertheless, IVH and SAH were not included in this study. A supervised multi-task aiding representation transfer learning network (SMART-Net) was proposed to overcome the complex training process of the current deep learning model and the inefficient prediction accuracy on the patient’s level (Kyung et al., 2022). Kwon et al. (2019) utilized a healthy brain template as auxiliary information for segmentation and employed U-Net to capture the difference between the input CT image and healthy template to segment ICH lesions more efficiently. The generative adversarial network (GAN) is also a common approach used in medical image segmentation tasks. A residual segmentation method with GAN (ReSGAN) was designed to learn a distribution of pseudo-normal brain CT scans and delineate the hemorrhaging areas (Toikkanen et al., 2021). To capture the interaction information between adjacent hematoma slices in CT images, Li et al. designed a slice expansion module and proposed two information transmission paths to expand the forward/backward slice respectively (Li X. Y. et al., 2022). The complicated annotation process of ICH lesions in CT images is one of the important factors which restricts the segmentation performance of the deep learning model. In order to make efficient utilization of unlabeled data, the semi-supervised learning approach such as the mean-teacher framework has also been transplanted by researchers for lesion segmentation (Cui et al., 2019).

However, the lesions of some hemorrhage types, such as SAH, are extremely extensive in the brain. The CNN-based methods which utilize local convolution kernel to obtain image features are difficult to effectively capture the long-distance dependencies in CT (Guo and Terzopoulos, 2021; Huang et al., 2022). Currently, the combination of the Transformer structure and CNN has been proven to be beneficial for capturing long-distance dependencies in images, which inspired us to use the Transformer structure for the segmentation of ICH lesions in this paper (Liu et al., 2020). In addition, the structure of the brain is roughly symmetrical (Liang et al., 2021). In a hemorrhagic stroke, this symmetry is commonly broken. Therefore, the symmetry change of brain structure can also be utilized for the segmentation of ICH lesions in CT images.

To solve the problem that the traditional CNN-based methods are difficult to capture the long-distance dependencies of CT images and the insufficient utilization of the symmetric structure of the brain, we proposed a novel deep learning method called symmetric Transformer network (Sym-TransNet) in this paper. The Sym-TransNet combines the Transformer structure with the traditional U-Net and adopts the symmetry prior knowledge in the network, which effectively improves the accuracy of segmentation and classification of ICH, reducing the workload of clinicians and providing a certain clinical basis for timely and accurate treatment of patients with ICH.

## Materials and methods

**FIGURE 1:**
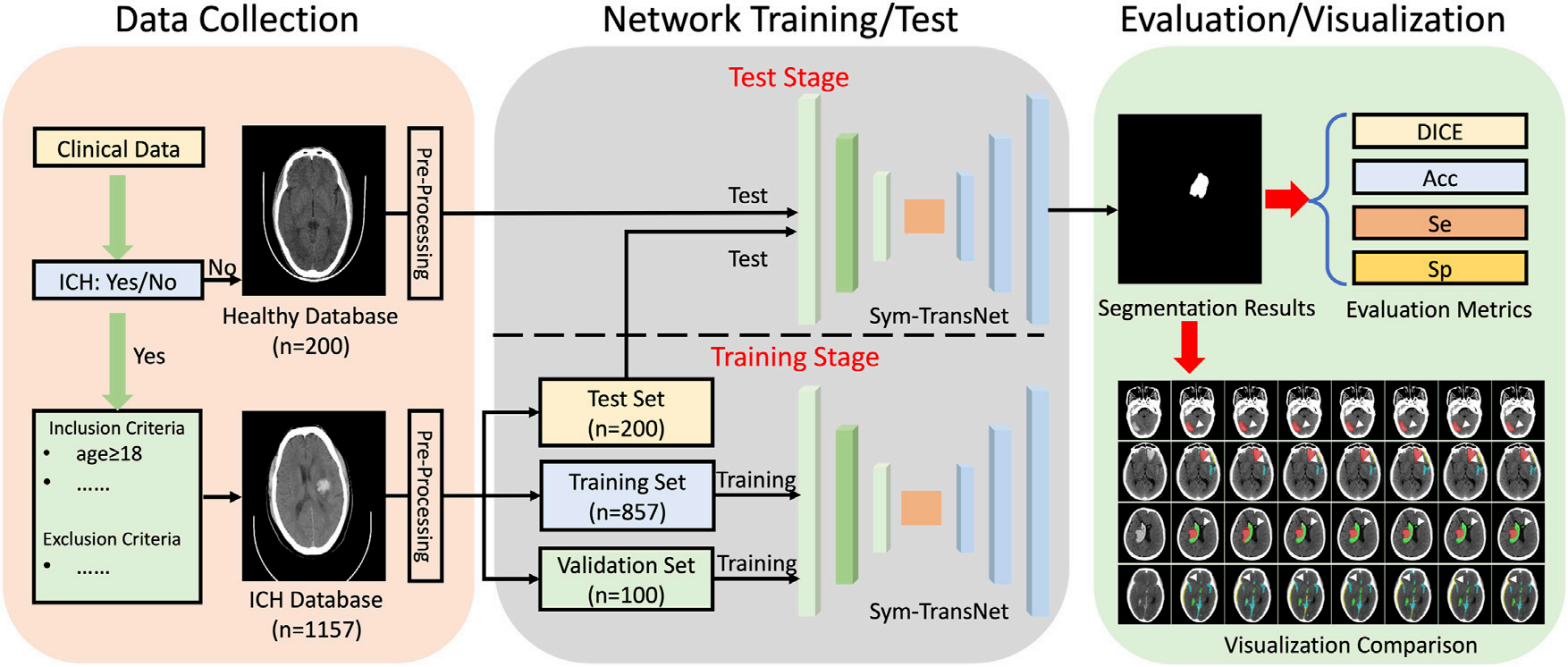
The workflow of the study.

In this retrospective study, a large number of CT images were collected clinically for training and testing of the proposed Sym-TransNet. Then we calculate the performance indicators of the proposed Sym-TransNet in the segmentation and diagnosis task of ICH, and visualize the results. The specific process is shown in Figure 1. Ethical approval for this study was waived by The Medical Ethics Committee of The First People’s Hospital of Kashi Prefecture because this study used anonymous data which was collected as part of routine diagnosis and treatment.

### Patients

A consecutive non-contrast head CT dataset, which retrospectively enrolled 1,157 patients who were diagnosed with ICH from January 2019 to April 2022 at the First People’s Hospital of Kashi Prefecture, was established in this study for the training, validation, and test of our proposed deep learning model. We developed three patient inclusion criteria as follows: 1) patients (age≥18 years) who were diagnosed with ICH; 2) the diagnoses coincided with non-contrast head CT scans and radiology reports; 3) the CT scans were performed within 3 days after onset of symptoms. In addition, we also integrated three exclusion criteria: 1) patients who refused to sign informed consent; 2) CT Scans with excessive motion/artifacts (image quality not suitable for ICH diagnosis); 3) patients with both hemorrhagic and ischemic strokes. In addition, CT scans of 200 healthy subjects were collected to evaluate the diagnostic specificity of our deep learning model. These CT scans were obtained during the physical examination in the First People’s Hospital of Kashi Prefecture from September 2019 to April 2022.

### Data collection and annotation

**FIGURE 2:**
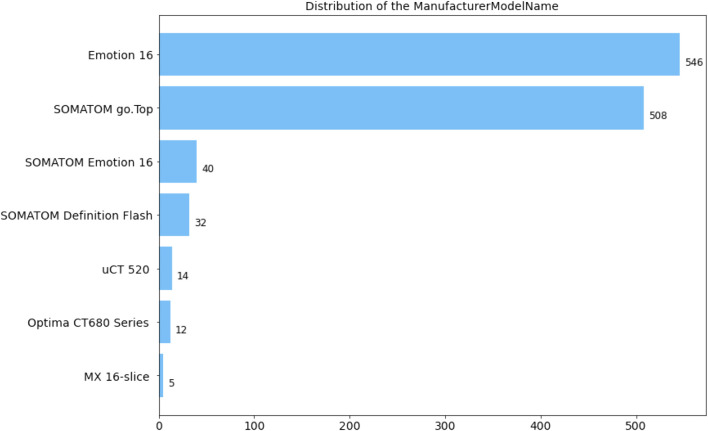
Distribution of CT information in the dataset. (a) Distribution of patient sex. O: unknown; F: female; M: male. (b) Distribution of the manufacturer model name.

All CT scans diagnosed with ICH utilized in this research were obtained by CT scanners produced by Siemens, Philips, United Imaging, and General Medical System. The slice thickness of the CT scans is mainly 5 mm. Specifically, the numerical distribution of the manufacturer model name in our ICH dataset is displayed in Figure 2. The dataset contained 678 male and 478 female patients with intracerebral hemorrhage, and the gender of one scan was unknown. In our ICH dataset, CT slices of 857 patients are adopted to train the deep learning model, CT slices of 100 patients are used to adjust parameters during the training stage, and CT slices of 200 patients are employed to evaluate the overall segmentation performance. After anonymizing sensitive information in original DICOM (Digital Imaging and Communications in Medicine) data, lesions in five sub-types of ICH, including IPH, IVH, EDH, SDH, and SAH, were annotated by six experienced radiologists. Based on the above stages, two senior neurologists with more than 5 years of experience corrected the mislabeling of preliminary lesion annotations and further refined the outline, location, and categories of hemorrhage lesions. The final segmentation gold standard was determined by senior neurologists after reaching a consensus.

### Data pre-processing

**FIGURE 3:**
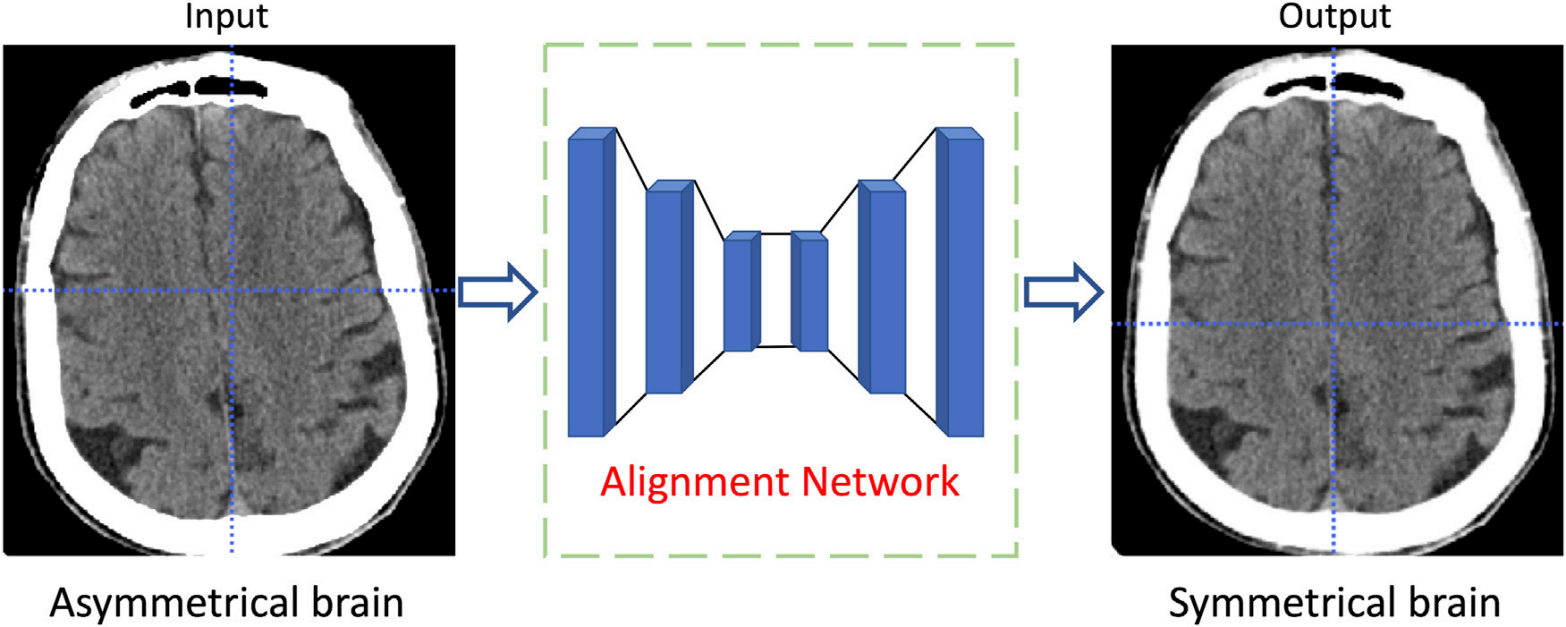
The diagram of the CT slice alignment through symmetric based alignment network.

To better adapt to the training stage of the deep learning network, we pre-process the CT images in our dataset. Specifically, the window level (WL)/window width (WW) of each CT image is set as 50/100 and normalized to have zero mean and unit variance (Yu et al., 2022). To reduce the consumption of computing resources, the size of each CT slice is resized to 512 × 512. In the test stage, all segmentation results obtained by the proposed model are upsampled to the original size for performance index calculation. Furthermore, some CT slices in the dataset are randomly flipped horizontally to increase the diversity of the training data. Due to the head position of the patient during the CT scan is not uniquely deterministic, the reconstructed brain structure in the CT slice is usually not horizontally symmetrical. To effectively utilize the medical prior knowledge that the brain structure is symmetrical, the symmetry-based alignment network (Wang et al., 2020) is utilized in this study to horizontally align the brain in all CT slices. As shown in Figure 3, the brain structure in the CT slice is transformed from asymmetric to horizontal symmetry after being processed by the alignment network.

### Symmetric prior knowledge-based deep learning model

**FIGURE 4:**
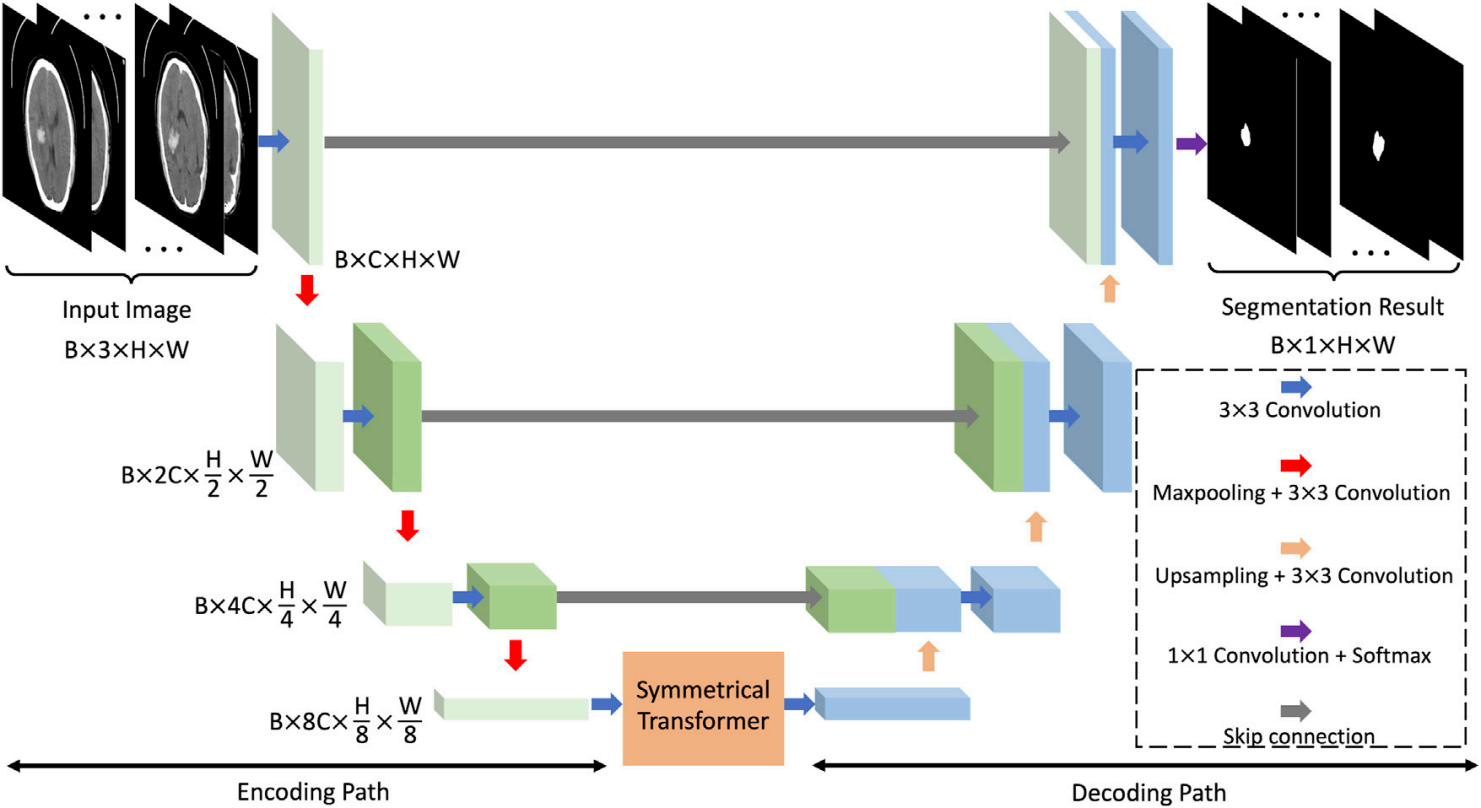
The framework of the proposed Sym-TransNet.

The convolutional neural network, which obtains high-level features in images through the convolution kernels and the down-sampling operation, is an efficient deep learning model utilized in various research fields. In the field of medical image segmentation, the U-Net is one of the most representative convolutional neural networks. The U-Net extracts and restores image features through interconnected codec paths, and has satisfactory performance in different segmentation tasks (Ibtehaz and Rahman, 2020). As shown in Figure 4, the proposed deep learning model is based on the U-Net framework, containing an image encoding path and an image decoding path. The bottom of the two paths is connected by a symmetric Transformer. We name this network the symmetric Transformer network (Sym-TransNet). The image preprocessed by the alignment network, which is regarded as the input of our Sym-TransNet, is transformed into a tensor of size B×C×H×W by the 3 × 3 convolution kernel, where *B* is the batch size which is pre-set in the training stage, *C* is the number of image channels, and *H* and *W* are the height and width of the image. In this paper, parameters B , C , H , and W are set to 64, 64, 512, and 512, respectively. The encoding path captures the high-level semantic information in CT images by using continuous 3 × 3 convolution and max-pooling and finally generates image features with the size of $B\times 8C\times \frac{H}{8}\times \frac{W}{8}$ .

**FIGURE 5:**
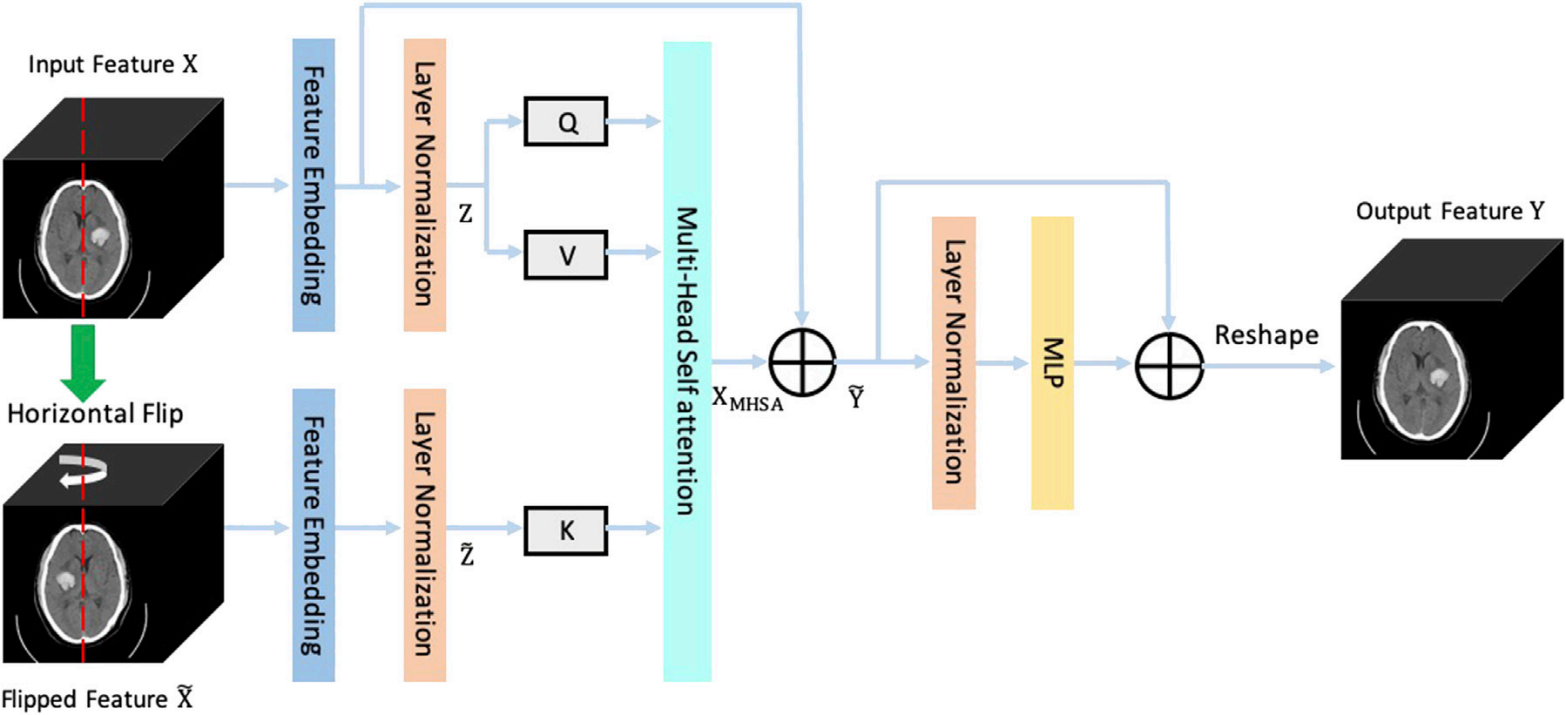
The diagram of the symmetrical Transformer.

The structure of the brain is roughly symmetrical. When a hemorrhagic stroke occurs, this symmetry is destroyed. Therefore, we can segment the bleeding area by capturing changes in symmetry in the brain CT image. However, the CNN which employs local convolution kernels is difficult to efficiently capture the long-distance symmetric relations in CT images. In recent years, the Transformer structure becomes a powerful approach for capturing long-distance dependencies in images and has achieved great success in computer vision tasks. Based on the medical prior knowledge that the brain structure is symmetrical, we propose a new Transformer structure named symmetrical Transformer to model long-distance symmetric relations in brain CT images. The structure of the proposed symmetrical Transformer is shown in Figure 5. We regard the image feature X obtained from the coding path as the input of the symmetric Transformer, and then flip it horizontally to get the flipped feature $\tilde{X}$ . The feature embedding and the layer normalization are employed to map the above two features into sequence features Z and $\tilde{Z}$ , which are both adapted to the Transformer structure. From Figure 5, the symmetrical Transformer mainly consists of two parts: the multi-head self-attention MHSA(∙) and multi-layer perceptron MLP(∙) . The input Q , V , and K of the MHSA can be calculated as follows:

$$\begin{array}{c} Q=ZW^{Q} \end{array}$$ (1)

$$\begin{array}{c} V=ZW^{V} \end{array}$$ (2)

$$\begin{array}{c} K={\tilde{\;Z}W}^{K} \end{array}$$ (3)

where the $W^{Q}$ , $W^{V}$ , and $W^{K}$ are learnable parameters, called a query transform matrix, a value transform matrix, and a key transform matrix, respectively. Further, the output of the MHSA $X_{MHSA}$ can be calculated as:

$$\begin{array}{c} X_{MHSA}=Softmax\left(\frac{QK^{T}}{\sqrt{d}}\right)V \end{array}$$ (4)

where d is a scaling factor that can solve the small gradient of the Softmax function. Thus, the output of the symmetrical Transformer Y can be represented as follows:

$$\begin{array}{c} Y=Reshape\left(MLP\left(\mathrm{LN}\left(\tilde{Y}\right)\right)+\tilde{Y}\right) \end{array}$$ (5)

$$\tilde{Y}=X_{MHSA}+Feature\;Embedding\left(X\right)$$ (6)

where Reshape(∙) , MLP(∙) , LN(∙) , and Feature Embedding(∙) denote image reshape operation, multi-layer perceptron, layer normalization, and feature embedding operation.

The features processed by the symmetrical Transformer are fed to the decoding path, and the image features are restored to the original resolution through continuous convolution operation and upsampling operation. Similar to the traditional U-Net, we adopt a skip connection between the encoding path and decoding path to obtain multi-scale features. Finally, 1 × 1 convolution and softmax function are utilized to generate the final segmentation result.

### Model training and evaluation metrics

The proposed Sym-TransNet is implemented based on PyTorch deep learning framework and is trained and evaluated on 4 NVIDIA RTX graphic cards. In the training stage, the Adam optimizer is adopted to optimize model parameters. In addition, cross-entropy and DICE loss functions are used to measure the distance between the segmentation results of the deep learning model and the golden standards during gradient backpropagation. We set the initial learning rate as $1\times 10^{-5}$ and the parameter weight decay as $1\times 10^{-3}$ , then utilize the exponential decay strategy to dynamically adjust the learning rate to avoid the local minimum. We set the maximum training epoch as 100, selecting the model with the best performance in the validation set to conduct performance evaluation on the test set.

In the test stage, the DICE coefficient is employed to evaluate the accuracy of the segmentation of ICH lesions. Assuming P is the prediction result of our proposed Sym-TransNet and G is the segmentation gold standard, the DICE coefficient can be calculated as:

$$\begin{array}{c} DICE=\frac{2\times \left|P\cap G\right|}{\left|P\cup G\right|} \end{array}$$ (7)

In addition to calculating the DICE coefficient of lesion segmentation, we also evaluate the accuracy (Acc), sensitivity (Sen), and specificity (Spe) of the proposed Sym-TransNet on the ICH diagnosis task. The diagnosis results are divided into four categories: true positive (TP), true negative (TN), false positive (FP), and false negative (FN). Thus, the Acc, Sen, and Spe can be calculated as follow:

$$\begin{array}{c} Acc=\frac{TP+TN}{TP+FP+TN+FN} \end{array}$$ (8)

$$\begin{array}{c} Sen=\frac{TP}{TP+FN} \end{array}$$ (9)

$$\begin{array}{c} Spe=\frac{TN}{TN+FP} \end{array}$$ (10)

### Statistical analysis

To statistically analyze the segmentation results obtained by the deep learning model, we utilized Wilcox rank-sum test to conduct pair-wise statistical tests (on DICE coefficient) between the proposed Sym-TransNet and several existing deep learning methods which are widely used in medical imaging (Wu et al., 2021). All of the statistical analysis in this paper was implemented in Python. We defined that the two methods were statistically different when the *p*-value <0.05.

## Results

### Satisfactory performance of Sym-TransNet for ICH lesion segmentation

After the parameters of the proposed Sym-TransNet are optimized on the training dataset, we evaluate the performance on the test dataset. To demonstrate that the proposed method has satisfactory segmentation performance compared with existing deep learning models, we faithfully reproduce several approaches commonly utilized in the field of medical image processing in recent years for comparison, including the U-Net, the U-Net with the dilated convolution (DU-Net) (Yu et al., 2017), the U-Net with SE block (SEU-Net) (Roy et al., 2019), the Dual-Attention Network (DA-Net) (Fu et al., 2019), and the High-Resolution Network (HR-Net) (Sun et al., 2019; Wang et al., 2021). The performance of the above methods in the segmentation of ICH lesions and the segmentation of five subtypes of ICH lesions is listed in Table 1. As shown in Table 1, our Sym-TransNet achieves an average DICE of 0.716, where the 95% confidence interval (95% CI) is 0.685–0.747, on the test dataset containing 200 patients with ICH, which is the best performance compared with the current methods in Table 1. Furthermore, for the segmentation of different subtypes of ICH lesions, the average DICE of IPH, IVH, EDH, SDH, and SAH by the Sym-TransNet is 0.784 (95% CI: 0.745–0.824), 0.680 (95% CI: 0.631–0.730), 0.359 (95% CI: 0.173–0.545), 0.534 (95% CI: 0.455–0.613), and 0.337 (95% CI: 0.293–0.382). In terms of IPH segmentation, the HR-Net is the model with the highest DICE among the comparison methods in Table 1, and our Sym-TransNet has improved by 0.26 on this basis. In the IVH case, the Sym-TransNet reaches the highest DICE score of all methods and 0.26 higher than the second-place method HR-Net. In addition, compared with other methods, the segmentation DICE coefficient of our method in EDH and SDH has been significantly improved. The reason why the performance of SDH and EDH is not as satisfactory as IPH and IVH are that the two kinds of bleeding lesions are close to the skull, resulting in the symmetry prior knowledge is not significantly beneficial to distinguishing the two kinds of lesions when they appear at the same time. As the lesions of SAH are very irregular in shape and often diffuse into the sulci, the traditional CNN methods based on local information modeling have low segmentation performance for SAH. The Sym-TransNet has improved the segmentation of SAH compared with the method based on CNN alone because of the combination of the Transformer structure that can capture the long-distance dependencies in the CT scan. The HR-Net is the best method among the compared methods for SAH segmentation because the multi-resolution method utilized in HR-Net captures more global information in SAH than other CNN-based methods. Compared with the HR-Net, the proposed Sym-TransNet further improves the DICE coefficient of 0.2 on SAH, indicating that the Transformer structure is effective in the segmentation of SAH.

**TABLE 1:** Segmentation performance of deep learning methods on ICH dataset.

Method	Parameters (1 × 10^6^)	ICH lesions (95% CI)	IPH (95% CI)	IVH (95% CI)	EDH (95% CI)	SDH (95% CI)	SAH (95% CI)
U-Net	2.47	0.624 (0.587, 0.661)	0.688 (0.638, 0.738)	0.518 (0.457, 0.580)	0.222 (0.083, 0.361)	0.321 (0.239, 0.404)	0.245 (0.206, 0.284)
DU-Net	4.83	0.611 (0.573, 0.649)	0.674 (0.622, 0.725)	0.496 (0.432, 0.560)	0.119 (0.014, 0.224)	0.256 (0.171, 0.341)	0.226 (0.186, 0.265)
SEU-Net	2.47	0.629 (0.592, 0.666)	0.691 (0.642, 0.741)	0.538 (0.478, 0.598)	0.253 (0.101, 0.405)	0.381 (0.304, 0.459)	0.256 (0.216, 0.295)
DA-Net	26.97	0.669 (0.636, 0.702)	0.739 (0.695, 0.782)	0.605 (0.550, 0.660)	0.244 (0.112, 0.375)	0.434 (0.348, 0.520)	0.269 (0.227, 0.311)
HR-Net	17.12	0.686 (0.656, 0.717)	0.758 (0.715, 0.802)	0.654 (0.603, 0.706)	0.205 (0.046, 0.364)	0.531 (0.458, 0.606)	0.317 (0.306, 0.389)
Ours	43.06	0.716 (0.685, 0.747)	0.784 (0.745, 0.824)	0.680 (0.631, 0.730)	0.359 (0.173, 0.545)	0.534 (0.455, 0.613)	0.337 (0.293,0.382)

**FIGURE 6:**
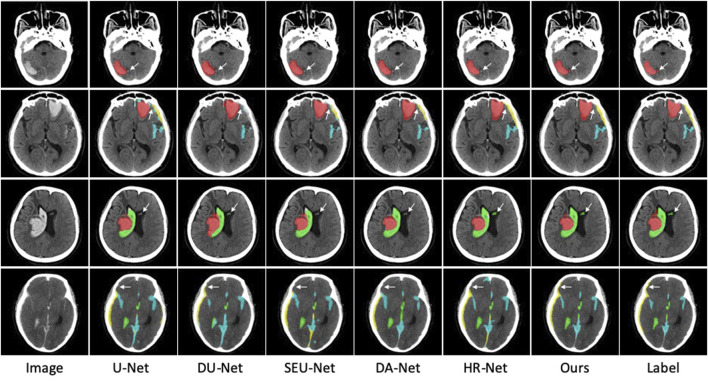
Visual comparison of segmentation results. The red, green, yellow, and blue pixels represent lesions of IPH, IVH, SDH, and SAH.

In addition to the DICE coefficient comparison, to more intuitively demonstrate the effectiveness of our method, the segmentation results of some ICH are also visualized in Figure 6. As far as the segmentation of cerebral hemorrhage lesions is concerned, the proposed Sym-TransNet can obtain the edges of lesions more consistent with manual labeling and can detect some small lesions missed by other methods. For IPH and IVH segmentation, when parenchymal hemorrhage breaks into the ventricle, the Sym-TransNet can more clearly define the interface between the two types of hemorrhage at the ventricle. For EDH and SDH, the Sym-TransNet reduces the risk of misidentifying the skull as bleeding. In addition, compared with other methods, our model can detect SAH diffusing in sulci more sensitively.

We also conduct pairwise statistical tests (between the Sym-TransNet and other methods) on the segmentation results of ICH lesions and five hemorrhage subtypes, as shown in Table 2. Except for EDH, the Sym-TransNet improves the segmentation performance of the U-Net, DU-Net, and SEU-Net with statistical significance (all *p*-values < 0.05). For DA-Net and Sym-TransNet, *p*-values were all less than 0.05 in ICH lesion, IPH, IVH, and SAH. For HR-Net, our Sym-TransNet improved ICH lesion segmentation significantly, but there was no statistical difference in the segmentation of the five bleeding subtypes.

**TABLE 2:** Statistical test results (*p*-value) between Sym-TransNet and other methods.

Methods types	ICH lesions	IPH	IVH	EDH	SDH	SAH
U-Net	2.95 $\times 10^{-5}$	1.09 $\times 10^{-5}$	4.63 $\times 10^{-5}$	2.6 $0\times 10^{-1}$	8.20 $\times 10^{-4}$	3.89 $\times 10^{-3}$
DU-Net	7.06 $\times 10^{-6}$	2.84 $\times 10^{-7}$	9.73 $\times 10^{-6}$	1.19 $\times 10^{-1}$	9.25 $\times 10^{-5}$	5.19 $\times 10^{-4}$
SEU-Net	9.71 $\times 10^{-5}$	3.69 $\times 10^{-5}$	2.33 $\times 10^{-4}$	2.25 $\times 10^{-1}$	5.86 $\times 10^{-3}$	9.30 $\times 10^{-3}$
DA-Net	8.22 $\times 10^{-3}$	4.01 $\times 10^{-4}$	1.62 $\times 10^{-2}$	2.36 $\times 10^{-1}$	9.08 $\times 10^{-2}$	2.61 $\times 10^{-2}$
HR-Net	4.04 $\times 10^{-2}$	1.17 $\times 10^{-1}$	3.41 $\times 10^{-1}$	3.55 $\times 10^{-1}$	8.29 $\times 10^{-1}$	7.72 $\times 10^{-1}$

### Transformer and symmetric prior knowledge improves the segmentation accuracy

**FIGURE 7:**
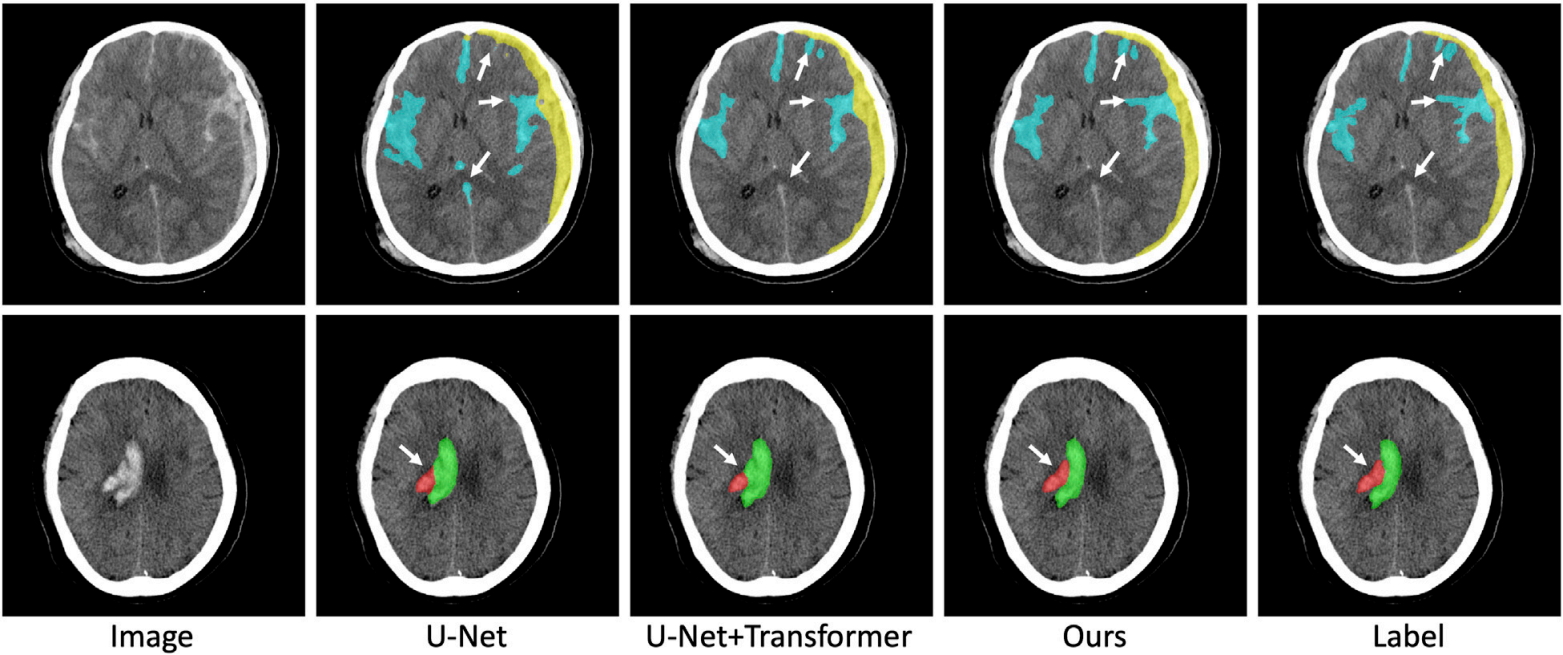
Visualization results of ablation experiments. The red, green, yellow, and blue pixels represent lesions of IPH, IVH, SDH, and SAH.

To prove the validity of the Transformer structure and symmetric prior knowledge utilized in this paper, we conduct ablation experiments for the Transformer structure and symmetric prior knowledge. The results of the ablation experiment are shown in Table 3. In the ablation experiment, the original U-Net is regarded as the baseline method, and the Transformer structure and symmetric prior knowledge are combined to observe the changes in the segmentation performance. As shown in Table 3, with the addition of the Transformer structure, the DICE coefficient of ICH lesions is significantly improved (from 0.624 to 0.691). In addition, the DICE coefficient of five different subtypes of ICH lesions is also increased (IPH: from 0.688 to 0.761, IVH: from 0.518 to 0.624, EDH: from 0.222 to 0.233, SDH: from 0.321 to 0.451, SAH: from 0.245 to 0.284). The improved segmentation performance of SAH lesions indicates that the combination of U-Net and Transformer structure is beneficial to the network to capture long-distance dependence in CT images and improve the segmentation accuracy of irregular lesions. Furthermore, after the fusion of symmetric prior knowledge, the segmentation performance of ICH lesions is improved (from 0.691 to 0.716). The segmentation DICE of the five subtypes also increase to different degrees (IPH increased by 0.023, IVH increased by 0.056, EDH increased by 0.126, SDH increased by 0.083, SAH increased by 0.053). The most obvious improvement is in the segmentation of IPH lesions because IPH contains abundant symmetric information (IPH lesions usually only appear on one side of the brain tissue), which indicates that the combination of symmetric information in the network is conducive to the improvement of the segmentation accuracy of ICH lesions. We visualize the results of the ablation experiment in Figure 7. It can be seen from Figure 7 that the combination of Transformer structure with U-Net results in clearer edge details of lesions and reduced part of false positive lesions. In particular, the Transformer structure improves the segmentation result of the deep learning model for SAH lesions which are widely distributed in the CT image, and reduces false positives in U-Net segmentation results, demonstrating that the Transformer structure can effectively capture the long-distance dependencies in the CT image. In addition, with the utilization of symmetric prior knowledge, the segmentation results of IPH and IVH are closer to the gold standard than the other two methods. As shown in Figure 7, the use of symmetric prior knowledge also refines the interface between IPH and IVH lesions, further improving the segmentation performance of the model.

**TABLE 3:** Ablation results of Sym-TransNet.

Method	ICH lesions (95% CI)	IPH (95%CI)	IVH (95%CI)	EDH (95%CI)	SDH (95%CI)	SAH (95%CI)
U-Net☑						
Transformer☒	0.624 (0.587, 0.661)	0.688 (0.638, 0.738)	0.518 (0.457, 0.580)	0.222 (0.083, 0.361)	0.321 (0.239, 0.404)	0.245 (0.206, 0.284)
Symmetric Prior☒						
U-Net☑						
Transformer☑	0.691 (0.659, 0.723)	0.761 (0.719, 0.802)	0.624 (0.569, 0.678)	0.233 (0.086, 0.379)	0.451 (0.370, 0.533)	0.284 (0.242, 0.326)
Symmetric Prior☒						
U-Net☑						
Transformer☑	0.716 (0.685, 0.747)	0.784 (0.745, 0.824)	0.680 (0.631, 0.730)	0.359 (0.173, 0.545)	0.534 (0.455, 0.613)	0.337 (0.293, 0.382)
Symmetric Prior☑						

### Sym-TransNet effectively diagnose patients with ICH

Clinically, the accurate diagnosis of ICH is of great significance for the follow-up treatment of patients. To demonstrate that the proposed deep learning model can sensitively detect patients with ICH while maintaining considerable specificity, we analyze the accuracy, sensitivity, and specificity of our model on 400 CT data (200 positive cases and 200 negative cases). Specifically, after the model obtains the segmentation results of the above 400 CT images, we determine whether the model classifies the corresponding data as ICH by calculating whether there are lesions in the segmentation results. Assuming that the data is a positive case and the segmentation result includes lesions, we record the segmentation result of this case as true positive (TP), and *vice versa* as false negative (FN). Assuming that the data is negative cases and there is no segmented lesion in the output of our model, we record the segmentation result of this case as true negative (TN), and otherwise as false positive (FP). As shown in Table 4, for the 200 positive samples, 197 cases are correctly diagnosed and only 3 cases are misdiagnosed as negative. Of the 200 negative samples, 168 are identified as negative and 32 were misdiagnosed as positive. Therefore, according to Eqs 8–10, we can calculate that the diagnostic accuracy, sensitivity, and specificity of the proposed model are 91.25%, 98.5%, and 84%, respectively. We also evaluate the diagnostic performance of the baseline method U-Net for ICH. The comparison of performance indexes between the two methods is shown in Table 5. Compared with the baseline method U-Net, the proposed Sym-TransNet has improved accuracy, sensitivity, and specificity (3%, 0.5%, and 5.5%, respectively), indicating that our method can effectively improve the overall performance of the ICH detection task based on the baseline model.

**TABLE 4:** Diagnostic results on CT data of 400 cases.

Total cases: 400 (positive: 200 negative: 200)	Positive (predict)	Negative (predict)
Positive (Actual)	197 (TP)	3 (FP)
Negative (Actual)	32 (FN)	168 (TN)

**TABLE 5:** Comparison of diagnostic performance of ICH.

Methods	Acc (%)	Sen (%)	Spe (%)
U-Net	88.25	98.00	78.50
Our Method	91.25	98.50	84.00

## Discussion

In this study, we verify that the proposed Sym-TransNet has better segmentation performance than the existing mainstream deep learning methods in the segmentation of ICH lesions and the segmentation of five ICH subtypes. Compared with the baseline model U-Net, the proposed deep learning model improves accuracy, sensitivity, and specificity in diagnosing ICH. Specifically, aiming at the five subtypes of ICH, the proposed Sym-TransNet achieves the highest DICE coefficient in intracerebral hemorrhage (comparison of DICE coefficients of five subtypes: IPH>IVH>SDH>EDH>SAH). Subdural hemorrhage and subarachnoid hemorrhage are the most easily missed or misdiagnosed subtypes of ICH due to irregular shape, unclear edge, and certain particularity of the site of hemorrhage. Subarachnoid hemorrhage is an example in which the lesions are filled with sulci, fissures, and cistern in casting shape, and the distribution is extensive and irregular. In addition, in the annotation process of segmentation gold standard, due to the existence of the CT volume effect, radiologists are difficult to accurately locate lesions of the subject who was diagnosed with EDH or SAH with less bleeding. The imprecise segmentation gold standard makes it difficult to train the deep learning model and directly affects its segmentation performance on the test set (the segmentation DICE coefficients of the model on EDH and SAH are 0.359 and 0.337, respectively). In order to alleviate the problems that exist in the process of annotation, many semi-supervised methods were proposed and have achieved better segmentation performance than fully supervised methods with a limited amount of labeled data. Therefore, the combination of the semi-supervised learning strategy may be an effective means to solve this problem, which is also our future research direction. In addition, researchers can also use the average value of multiple professional physicians as the final gold standard to alleviate the impact of inaccurate labeling on segmentation performance.

The segmentation performance of Sym-TransNet on IPH and IVH is significantly improved compared with existing methods. For the above two types of ICH, Sym-TransNet improved the DICE coefficient by 0.26 on the basis of the HR-Net. In terms of visualization of segmentation results, the segmentation results obtained by Sym-TransNet are more consistent with manual annotation, and some microscopic lesions missed by the other methods are detected. When IPH invades the ventricle, Sym-TransNet can more clearly distinguish the interface between the two types of hemorrhage. For EDH and SDH, Sym-TransNet reduced the risk of misidentifying the skull as a bleeding point. Moreover, compared with other deep learning methods, our model is more sensitive to detecting the SAH lesions in the sulci. Therefore, the proposed Sym-TransNet can more effectively segment different types of ICH lesions from CT images of ICH patients, which has potential clinical application prospects.

However, this study also has some limitations. From the perspective of data collection, as a retrospective study, selection bias may exist in this paper. Although a large number of CT data were included as the training set in this study, the sample size of the test set was insufficient, which probably results in accidental segmentation performance. Therefore, it is necessary to increase the test sample size to verify the model performance. Additionally, all of our CT scans came from a single center, and the diversity of samples can be further improved. From the perspective of the number of trainable parameters for deep learning models, the proposed Sym-TransNet is not optimal. We list the number of parameters for several mainstream models used for performance comparisons in this paper in Table 1. As shown in Table 1, Sym-TransNet has the highest number of model parameters. This is because the self-attention mechanism in the Transformer model requires a large amount of computation to obtain the long-distance dependence information. In the future, we will explore a more lightweight Transformer model for ICH lesion segmentation, which will be better applied in clinical practice.

In summary, Sym-TransNet proposed in this paper can accurately segment the ICH lesions and the five hemorrhage subtypes, improving the performance on the basis of the U-Net for the diagnosis of ICH. Sym-TransNet is expected to help relieve the workload of radiologists and reduce the rate of misdiagnosis of ICH in clinical practice, providing a basis for assisting clinical decision-making.

## Data Availability

The raw data supporting the conclusion of this article will be made available by the authors, without undue reservation.
